# Association between infection and fever in terminations of pregnancy using misoprostol: a retrospective cohort study

**DOI:** 10.1186/s12884-016-1188-1

**Published:** 2017-01-05

**Authors:** Tobias A. J. Nijman, Kevin G. J. A. Voogdt, Pim W. Teunissen, Patrick J. (JP) van der Voorn, Christianne J. M. de Groot, Petra C. A. M. Bakker

**Affiliations:** 1 Department Obstetrics and Gynecology, Division Woman & Baby, UMC, Lundlaan 6, 3508 AB Utrecht, The Netherlands; 2 Department Obstetrics and Gynecology, VUmc, Amsterdam, The Netherlands; 3 Department Pathology, VUmc, Amsterdam, The Netherlands

**Keywords:** Misoprostol, Fever, Termination of pregnancy, Abortion, Infection

## Abstract

**Background:**

Fever is a well-known side effect of misoprostol, but clinically difficult to distinguish from an intra uterine infection. The aim of this study was to determine the incidence of fever in terminations of pregnancy (TOP) using misoprostol and to evaluate fever as indication of intra uterine infection.

**Methods:**

A retrospective cohort study was performed. Consecutive second trimester TOP with misoprostol between January 2008 and October 2012 were selected. We included 403 cases and determined the incidence of fever. To examine intra uterine infection as plausible cause of fever, pathological examination reports of placentas were reviewed for signs of infections.

**Results:**

The incidence of fever was 42%. Logistic regression showed a dose dependent association between dosage misoprostol and degree of fever (OR 1.86; 95% CI: 1.3–2.7). There was no association between fever and epidural analgesia. Fever has a sensitivity of 55% and a specificity of 58% as a marker of intra uterine infection. The positive predictive value of fever for an intra uterine infection is 4% and the negative predictive value is 98%.

**Conclusion:**

Administration of misoprostol for the indication TOP is strongly associated with fever during labor. Fever is a poor predictor of intra uterine infection in the context of TOP.

## Background

Termination of pregnancy (TOP) in case of intra uterine fetal death (IUFD) or in pregnancies complicated by congenital abnormalities is a frequently practiced procedure worldwide [1]. Worldwide, every year 22% of all pregnancies (46 million) result in an induced abortion; this includes both medical and surgical methods [1, 2]. Most terminations of pregnancy are performed before 12 weeks of gestation and are mostly performed by dilatation and curettage. Yet approximately 10 to 15% are performed in the second trimester, defined as 12 to 28 weeks of gestation, and are mostly conducted medically. These second trimester TOPs are responsible for two-thirds of all major complications in TOPs, including severe haemorrhage and infection [1, 3] In addition, the number of second trimester TOPs has increased since the introduction of prenatal screening programs following an increase in detection rate and earlier detection of fetal anomalies [1, 2].

The most efficient regimen for a medical TOP is a combination of mifepristone, an antiprogesterone, and vaginally administered misoprostol, a synthetic prostaglandin E1 analogue [4]. Misoprostol was originally developed for prevention and treatment of peptic ulcer disease. A side effect of misoprostol is uterine contractility and cervical ripening which has led to its off-label use for TOP [1, 5]. Fever is a well-described side effect of misoprostol [1, 3, 5]. The incidence of fever as a side effect of misoprostol reportedly varies from 10 up to 50% [1, 5–11]. The majority of fever is temporary and resolves spontaneously. However, clinically, fever as a side effect of misoprostol and fever caused by intra uterine infection can be difficult to distinguish. Another explanation for the development of fever after administration of misoprostol is that prostaglandin interferes with the hypothalamus (where the body temperature is regulated) by a shift in the temperature set point, which results in a systemic response of increased temperature [12].

Recent research showed that clinical signs of an infection are not accurate in the diagnosis intra uterine infection in term parturients [13]. The significance of fever as an indicator of intra uterine infection in women undergoing TOP has never been addressed in literature. The increasing incidence of epidural analgesia during labour, which is also associated with fever, makes this distinction even more difficult [14]. One of the consequences of incorrectly interpreting fever as a sign of infection is the unnecessary administration of antibiotics adding to the growing problem of bacterial resistence [15].

## Aim of the study

The primary aim of this study is to determine the incidence and evaluate the test characteristics of fever in second trimester TOP using misoprostol for the prediction of intra uterine infection.

## Methods

### Study design

A retrospective descriptive study was performed at the VU University Medical Center, Amsterdam, the Netherlands, a tertiary academic center. All consecutive second trimester terminations of pregnancy (12–28 weeks gestation) using misoprostol occurring between January 2008 and October 2012 were selected.

Inclusion criteria were defined as a second trimester TOP (gestational age between 12 and 28 weeks) by misoprostol using the local second trimester regimen. Exclusion took place for the following reasons: (1) fever or antibiotic therapy before starting misoprostol or suspected infection before induction, (2) incomplete medical charts (e.g. no temperatures noted) or (3) protocol violation in misoprostol dosage.

The VUMC second trimester protocol for TOP, which is based on the national guidelines of the Dutch Society of Obstetrics and Gynaecology consists of 200 mg mifepristone orally 36–48 h before starting misoprostol. The national guidelines are based on guidelines by the Royal College of Obstetricians and Gynaecogists and the World Health Organisation. At the day of induction, a start dose of 600 μg is administered vaginally, in the posterior fornix, followed by 400 μg every 3 h, with a maximum of 5 doses per day. Patient’s vital functions are being measured at least every 8 h: temperature, heart rate and blood pressure.

Fever was defined as a rectal temperature >37.8 °C (100 °F). The following information was collected for all women (with and without fever). Vital parameters, used as indicators of shock, were listed: lowest systolic and diastolic blood pressure (mmHg) and highest heart rate (bpm). When antibiotics were administered, the initiation time and duration of therapy were noted. Blood samples and cultures are not drawn routinely, but when fever is present it is at the clinicians discretion to decide which diagnostics are being done. Infection parameters in the blood such as C-reactive protein (CRP) and leucocytes were noted. The normal range of CRP was established as a value < 10 mg/L, the normal range of leucocytes in pregnancy as 5.6–13.8*10^9/L [16, 17]. The number of patients with fever, total dose of misoprostol and induction-to-expulsion time were determined. To investigate an infection as a probable cause for fever, results of blood, vagina or urine cultures were reviewed. In addition, reports of pathological examination of the placentas from all women have been reviewed for signs of infections. If a report mentioned signs of infection, histologic placental material was re-examined by an anatomical pathologist, specialized in placental pathology (JPvdV).

The placentas with signs of infection were categorized in stages of inflammatory response. Stage II or III reaction are indicative of a clinical intra uterine infection [18].

Stage:

- 0. No sign of infection
- I. Early: acute subchorionitis or chorionitis
- II. Intermediate: acute chorioamnionitis
- III. Advanced: necrotizing chorioamnionitis

Grade:

- 0. None
- I. Mild-moderate
- II. Severe

### Ethical approval

Due to the retrospective nature of this study ethical approval was not required.

### Legality

Legality of terminations of pregnancy has the same principles as abortions for social indications in the Netherlands. Termination of pregnancy, for either indication, is legal before 24 weeks of gestation taken into consideration a 5 day-period of reflection. After 24 weeks TOP is only permitted in cases where fetal malformation are incompatible with life or severe morbidities are expected.

### Statistical analysis

Non-parametric tests were used because our data were not normally distributed. Using presence of stage II or III reaction as the gold standard for an intra uterine infection, test characteristics of fever could be calculated.

The Mann-Whitney U-test was for continues variables. We categorized gestational age (GA) (>98 days, 98–112 days, 112–126 days, 126–140 days, 140–154 days, 154–168 days and >168 days) and the administered doses of misoprostol (600 μgr, 600–1200 μgr, 1200–1800 μgr, 1800–2400 μgr, 2400–3000 μgr and ≥3000 μgr). The Kruskal-Wallis-test was used to compare the differences in mean highest temperature in categorized dose of misoprostol and gestational age. We used the Chi-Square test or Fisher’s exact test for categorical variables. To control for the confounder epidural analgesia, a multiple logistic regression with the presence of fever as dependent was performed. Determinants were presence of epidural analgesia and required dose of misoprostol. We expected a strong association between duration of labor and required dosage of misoprostol. Pearson correlation was calculated to verify this. Results are presented in Odds Ratio (OR) and 95% Confidence Interval (CI). Tests were considered statistically significant when *p* < 0.05. Statistical analysis was performed using SPSS (SPSS inc., version 20, Chicago, USA).

## Results

In the period January 2008 till October 2012, 458 terminations of pregnancy took place. A total of 403 women were included. Women were excluded for the following reasons: (1) fever before induction of labor (*n* = 11), (2) incomplete medical charts (*n* = 30) or (3) protocol violation of misoprostol dosage (*n* = 14).

Of all women, 168 (42%) developed fever. The majority of these patients (71.5%) had a rectal temperature between 38.0 and 38.9 °C. Most fever developed within 10 h after the start of induction and disappeared after expulsion. Compared to those who did not develop fever, women with fever more often were of advanced gestational age, more often were nulliparous, had a longer induction-expulsion time, and a higher total dosage misoprostol (Table 1).

**Table 1:** Patient characteristics

	Patients with fever (*n* = 168)	Patients without fever (*n* = 235)	*P*-value
Gestational age in days (Mean ± SD)	139 ± 58	121 ± 27	<0.001
Nulliparous, n (%)	63.1	39.1	<0.001
Induction-expulsion time, hours (Mean ± SD)	15 ± 14	8 ± 6	<0.001
Birth weight, grams (Mean ± SD)	365 ± 319	208 ± 227	<0.001
Total administered dose of misoprostol,μgr (Mean ± SD)	1946 ± 1101	1254 ± 611	<0.001
Total blood loss, mL (Mean ± SD)	313 ± 416	382 ± 444	0.397
Indications for TOP			0.10
Fetal anomaly, n (%)	154 (91.7%)	202 (85.9%)	
IUFD, n (%)	11 (6.5%)	19 (8.1%)	
Miscellaneous n (%)	3 (1.8%)	14 (6.0%)	
Vital parameters (mean ± SD)			
Systolic blood pressure, mmHg	109 ± 12	105 ± 12	<0.01
Diastolic blood pressure, mmHg	66 ± 10	65 ± 10	0.161
Heart rate, beats per minute	83 ± 13	76 ± 10	<0,001
CRP values	*n* = 45	*n* = 5	0.21
Elevated CRP values, n (%)	14 (31%)	3 (60%)	
Leukocyte values	*n* = 45	*n* = 5	0.26
Elevated leukocyte values, n (%)	22 (49%)	3 (60%)	
non parametric tests were used in case of not normally distributed data			
Type of culture	*n*	Positive, *n* (%)	Negative, *n* (%)
Blood	18	0 (0)	18 (100)
Vaginal	19	3 (16)	16 (84)
Urine	12	0 (0)	12 (100)

### Pathological examination of the placentas

In total, 298 out of 403 (74%) placenta reports could be retrieved. In patients with fever 127 (76.0%) placentas were sent for examination. In patients without fever 171 (72.5%) placentas were sent for examination. In patients with fever, 5 placentas (3%) showed a stage II or III reaction, which is indicative for an intra uterine infection [18]. In patients without fever, 4 patients (2%) were found to have a stage II or III reaction. Table 2 shows the characteristics of women in which the placenta showed signs of stage II or III infection.

**Table 2:** Characteristics of women with infected placentas

Parity	GA	Dose misoprostol (μgr)	Stage of infection	Temp (°C)	CRP (mg/L)	Leukocytes (*10^9/L)	Culture	EDA	Duration of labour	Indication
0	17 + 0	6800	III	39	13,8	10,8	V: neg	Yes	71h30m	Trisomy 21
0	17 + 5	1000	II	38,5	-	-	-	No	05m45m	IUFD
0	21 + 0	2200	II	38,4	-	-	-	No	15h50m	Increased nuchal translucency
1	19 + 1	1000	II	37,9	-	-	-	No	06h45m	IUFD
3	24 + 1	1800	III	38,1	22	13,5	V/U: neg	No	12h00m	Immature rupture of membranes
2	14 + 5	1000	III	<37,8	-	-	-	Yes	05h32m	Immature rupture of membranes
0	15 + 0	1000	II	<37,8	-	-	-	Yes	07h30m	Hychroma colli
0	20 + 0	1000	II	<37,8	-	-	-	No	00h10m	Immature rupture of membranes
1	23 + 4	1000	III	<37,8	21	15,6	-	No	04h10m	Immature rupture of membranes

*GA* gestational age, *CRP* C-reactive protein, *EDA* epidural analgesia, *IUFD* intra uterine fetal death

Culture: *v* vaginal, *u* urine, *Neg* negative

### Test characteristics of fever

Using presence of stage II or III reaction as the gold standard for an intra uterine infection, test characteristics of fever were calculated. Table 3 shows the test characteristics (sensitivity, specificity, positive predictive value and negative predictive value), based on the group for which revision of pathology reports was available. Fever has a sensitivity of 55% and a specificity of 58%. The positive predictive value for an infection is 4% and the negative predictive value is 98%.

**Table 3:** Test characteristics of fever

	Infection +	Infection -	Total	
Fever +	5	122	127	PPV: 4%
Fever -	4	167	171	NPV: 98%
Total	9	289	298	
	Sensitivity: 55%	Specificity: 58%		

+: present; -: absent; *PPV* positive predictive value, *NPV* negative predictive value

### Dose response relation

**Fig. 1:**
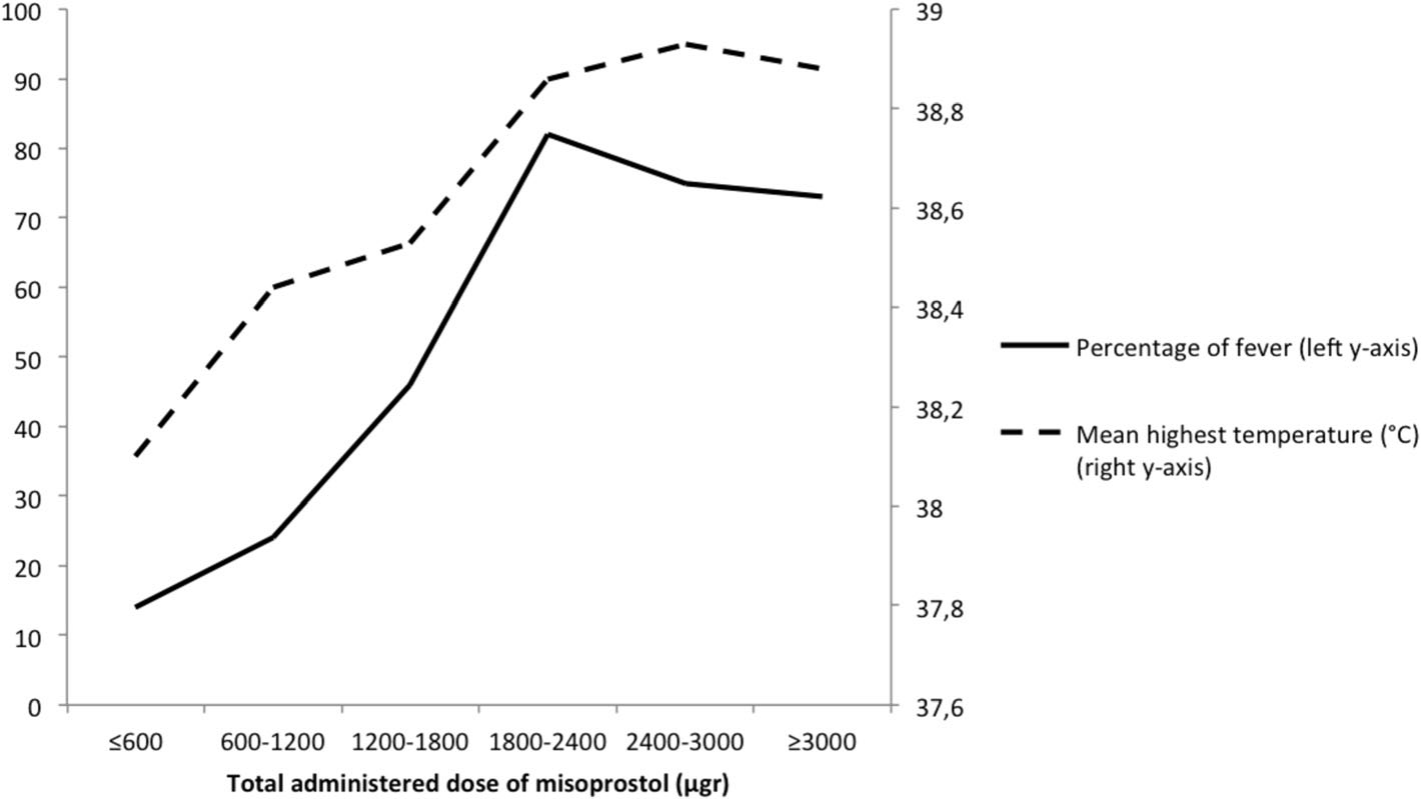
Administered doses of misoprostol with corresponding proportions of fever and the mean highest temperature

The mean administered dose of misoprostol in the group of patients with fever was significantly higher than in the group without fever (Table 1). Fig. 1 shows the increasing temperature and percentage of fever with increasing dosage of misoprostol. Pearson correlation showed a strong association (0.919; *p* < 0.001) between required dose of misoprostol and duration of induction. For analgesia during induction, 36.7% received epidural analgesia. 61,5% of those patients had fever, which is significantly higher than in the group without epidural analgesia. However, binary logistic regression showed no significant effect of epidural analgesia (OR 0.32; 95% CI: 0.07–1.37). The effect of misoprostol on the presence of fever proved to be significant (OR 1.86; 95% CI: 1.3–2.7). The mean administered dose of misoprostol was significant higher in the group with epidural analgesia (*p* < 0.01).

### Antibiotic therapy

In 31 (19%) of patients with fever antibiotic therapy was started. Grade 2 or higher chorioamnionitis was found in two of these patients. So in two out of 31 women an intra uterine infection was found. In the first case, antibiotic therapy was started directly after expulsion of the fetus because of smelly discharge. Vaginal culture showed no growth of bacteria. The second case presented with immature rupture of membranes. Antibiotic therapy was started at the beginning of induction before fever was noted. Vaginal swab also was negative.

## Discussion

We found an incidence of 42% of fever in terminations of pregnancy using misoprostol. Based on placenta pathology as the gold standard for defining intra uterine infection, fever had a positive predictive value of 4% in our study population, rendering it useless as a sign for the presence of an intra uterine infection. Our results show that in the majority of cases the presence of fever appears to be a side effect of misoprostol rather than a sign of an intra uterine infection. Furthermore a dose response relation was found, which makes the evidence of fever as a side effect of misoprostol stronger.

Fever is a well described side effect of misoprostol [1, 3, 5]. The mechanism of fever after administration of misoprostol, a Prostaglandin E1, has been investigated for the indication prevention of postpartum hemorrhage [12, 19]. Prostaglandins E-series are involved in the endogenous origin of fever by interfering with the hypothalamus, where body temperature is regulated, inducing a shift in the hypothalamic set point by interaction resulting in a systemic response. Due to this response, heat-creating effects are initiated to achieve increased temperature level [12]. On the other hand, fever is known to be associated with the presence of an infection. Infection is a serious maternal complication resulting in 15% of maternal death worldwide [20]. This underlines the importance of discriminating the cause of fever; drug induced or underlying intra uterine infection. The urge is for a sensitive test during labor. Since pathological conformation is the gold standard for intra uterine infection, we used the diagnosis only to confirm after delivery and to test its sensitivity. Table 1 describes the culture results. Only three out of 19 vaginal cultures turned out positive. One was positive for gardnerella vaginalis and candida albicans, which are not a likely cause of fever [21, 22]. The other showed positive for group B streptococcus (GBS). In 19% of pregnant women, GBS can be found in vaginal culture. Most of these women are asymptomatic [23]. Furthermore placentas of both patients with positive vaginal cultures did not show signs of infection.

The strength of this study is that one specialized pathologist reviewed all placentas. This ensures the uniformity of the examination and minimized the risk of intra observer variability. Furthermore all patients received the same regimen of misoprostol. This increases the comparability of the patients. A limitation of this study is that it is retrospective. Therefore the incidence of fever might be underreported; the temperature was not measured every hour and occasionally a peak temperature might have been missed. However, we used strictly rectal temperatures for a high uniformity in the measurements. Furthermore, due to the retrospective nature of the study not all data required was present. Preferably we would have cultures, blood examination and placental examination from all patients. A second limitation is that not all placentas were investigated (*n* = 105). However the amount of placentas investigated did not differ between women with and without fever. Even if all placentas not investigated would have showed signs of stage II or III infection fever would have had a sensitivity of 43% and a specificity of 58%. The positive predictive value for an infection would have been 27% and the negative predictive value 71%. So also in this hypothetical calculation fever is not an accurate predictor of an intra uterine infection.

In the majority of the cases the presence of fever seems to be a side effect and not a sign of an intra uterine infection. There were no patients in septic shock or critically ill in this study population. As in most cases fever developed within 10 h after the start of induction and disappeared after expulsion, it might be sufficient to only administer antipyretic drugs such as paracetamol, and IV-fluids. The use of antibiotics during termination of pregnancy using misoprostol is therefore debatable. Given the increased levels of antibiotic resistance [14], it is recommendable to be cautious with initiating antibiotic therapy until other clinical signs of infection are more likely. Future research should focus on prospective data collection of women with a second trimester TOP using misoprostol, with placental histopathological examination of all placentas, blood samples and cultures. Recent research has shown that procalcitonin is a useful predictor for bacterial infection [24]. It could be interesting to investigate whether procalcitonin can also be useful in the prediction of intra uterine infection in patients with second trimester TOP using misoprostol. Combining all data might lead to development of a prediction model for the presence of a intra uterine infection.

## Conclusion

In conclusion administration of misoprostol for the indication TOP is highly associated with fever during labor, but fever has no accurate test characteristics for the presence of an intra uterine infection.

## Abbreviations

CI: Confidence interval
CRP: C-reactive protein
GA: Gestational age
GBS: Group B streptococcus
IUFD: Intra uterine fetal death
OR: Odds ratio
TOP: Termination of pregnancy

## References

[1] Lalitkumar S, Bygdeman M, Gemzell-Danielsson K (2007). Mid-trimester induced abortion: a review. Hum Reprod Update.

[2] World Health Organization (2012). Safe abortion: technical and policy guidance for health systems.

[3] Ho PC, Blumenthal PD, Gemzell-Danielsson K, de Gomez Ponce LR, Mittal S, Tang OS (2007). Misoprostol for the termination of pregnancy with a live fetus at 13 to 26 weeks. Int J Gynaecol Obstet.

[4] Wildschut H, Both MI, Medema S, Thormee E, Wildhagen MF, Kapp N. Medical methods for mid-trimester termination of pregnancy. Cochrane Database Syst Rev. 2011;(1):CD005216. doi:10.1002/14651858.CD005216.pub2. Review. 10.1002/14651858.CD005216.pub2 PMC8557267 21249669

[5] Pongsatha S, Tongsong T (2011). Outcomes of pregnancy termination by misoprostol at 14-32 weeks of gestation: a 10-year-experience. J Med Assoc Thai.

[6] Ebbers S, Creemers JW, Lotgering FK (2009). [Termination of pregnancy in the 2nd trimester: mifepriston/misoprostol preferable to sulprostone]. Ned Tijdschr Geneeskd.

[7] Wong KS, Ngai CS, Yeo EL, Tang LC, Ho PC (2000). A comparison of two regimens of intravaginal misoprostol for termination of second trimester pregnancy: a randomized comparative trial. Hum Reprod.

[8] Ghorab MN, El Helw BA (1998). Second-trimester termination of pregnancy by extra-amniotic prostaglandin F2alpha or endocervical misoprostol. A comparative study. Acta Obstet Gynecol Scand.

[9] Chong E, Tsereteli T, Nguyen NN, Winikoff B (2012). A randomized controlled trial of different buccal misoprostol doses in mifepristone medical abortion. Contraception.

[10] Ngai SW, Tang OS, Ho PC (2000). Randomized comparison of vaginal (200 microg every 3 h) and oral (400 microg every 3 h) misoprostol when combined with mifepristone in termination of second trimester pregnancy. Hum Reprod.

[11] Brouns JF, van Wely M, Burger MP, van Wijngaarden WJ (2010). Comparison of two dose regimens of misoprostol for second-trimester pregnancy termination. Contraception.

[12] Curtin WM, Katzman PJ, Florescue H, Metlay LA (2013). Accuracy of signs of clinical chorioamnionitis in the term parturient. J Perinatol.

[13] Jones L, Othman M, Dowswell T, Alfirevic Z, Gates S, Newburn M (2012). Pain management for women in labour: an overview of systematic reviews. Cochrane Database Syst Rev.

[14] Davey P, Brown E, Charani E, Fenelon L, Gould IM, Holmes A, Ramsay CR, Wiffen PJ, Wilcox M. Interventions to improve antibiotic prescribing practices for hospital inpatients. Cochrane Database Syst Rev. 2013;(4):CD003543. doi:10.1002/14651858.CD0003543.pub3. Review. 10.1002/14651858.CD003543.pub3 23633313

[15] Kushner I (1982). The phenomenon of the acute phase response. Ann N Y Acad Sci.

[16] Tzur T, Weintraub AY, Sergienko R, Sheiner E. Can leukocyte count during the first trimester of pregnancy predict later gestational complications? Arch Gynecol Obstet. 2013;287(3):421-7. doi:10.1007/s00404-012-2603-0. 10.1007/s00404-012-2603-0 23080549

[17] Redline RW, Faye-Petersen O, Heller D, Qureshi F, Savell V, Vogler C (2003). Amniotic infection syndrome: nosology and reproducibility of placental reaction patterns. Pediatr Dev Pathol.

[18] Durocher J, Bynum J, Leon W, Barrera G, Winikoff B (2010). High fever following postpartum administration of sublingual misoprostol. BJOG.

[19] Elati A, Weeks A (2012). Risk of fever after misoprostol for the prevention of postpartum hemorrhage: a meta-analysis. Obstet Gynecol.

[20] The World Health Report 2005: Make every mother and child count. 2005. Ref Type: Generic. ISBN 9241562900.

[21] Klebanoff MA, Schwebke JR, Zhang J, Nansel TR, Yu KF, Andrews WW (2004). Vulvovaginal symptoms in women with bacterial vaginosis. Obstet Gynecol.

[22] Lamont RF, Sobel JD, Akins RA, Hassan SS, Chaiworapongsa T, Kusanovic JP (2011). The vaginal microbiome: new information about genital tract flora using molecular based techniques. BJOG.

[23] Valkenburg-van den Berg AW, Houtman-Roelofsen RL, Oostvogel PM, Dekker FW, Dorr PJ, Sprij AJ (2010). Timing of group B streptococcus screening in pregnancy: a systematic review. Gynecol Obstet Invest.

[24] de Jong E, van Oers JA, Beishuizen A, Vos P, Vermeijden WJ, Haas LE, Loef BG (2016). Efficacy and safety of procalcitonin guidance in reducing the duration of antibiotic treatment in critically ill patients: a randomised, controlled, open-label trial. Lancet Infect Dis.

